# Multifunctional miR-155 Pathway in Avian Oncogenic Virus-Induced Neoplastic Diseases

**DOI:** 10.3390/ncrna5010024

**Published:** 2019-03-13

**Authors:** Megha Sravani Bondada, Yongxiu Yao, Venugopal Nair

**Affiliations:** 1 Avian Oncogenic Viruses, The Pirbright Institute and the UK-China Centre of Excellence for Research on Avian Diseases, Pirbright, Ash Road, Guildford, Surrey GU24 0NF, UK; megha.bondada@pirbright.ac.uk (M.S.B.); yongxiu.yao@pirbright.ac.uk (Y.Y.); 2 Department of Zoology, University of Oxford, 11a Mansfield Road, Oxford OX1 3SZ, United Kingdom.

**Keywords:** miR-155, oncogenesis, EBV, KSHV, MDV, ALV, REV

## Abstract

MicroRNAs (miRNAs) are small noncoding RNAs that fine-tune the responses of the cell by modulating the cell transcriptome and gene expression. MicroRNA 155 (miR-155) is a conserved multifunctional miRNA involved in multiple roles including the modulation of the immune responses. When deregulated, miR-155 can also contribute to cancer as has been demonstrated in several human malignancies such as diffuse large B cell lymphoma, chronic lymphocytic leukemia, as well as in Epstein–Barr virus (EBV)-induced B cell transformation. Avian oncogenic viruses such as Marek’s disease virus (MDV), avian leukosis virus (ALV), and reticuloendotheliosis virus (REV) that account for more than 90% of cancers in avian species, also make use of the miR-155 pathway during oncogenesis. While oncogenic retroviruses, such as ALV, activate miR-155 by insertional activation, acutely transforming retroviruses use transduced oncogenes such as v-*rel* to upregulate miR-155 expression. MDV on the other hand, encodes a functional miR-155 ortholog mdv1-miR-M4, similar to the miR-155 ortholog kshv-miR-K11 present in Kaposi’s sarcoma-associated herpesvirus (KSHV). We have shown that mdv1-miR-M4 is critical for the induction of MDV-induced lymphomas further demonstrating the oncogenic potential of miR-155 pathway in cancers irrespective of the diverse etiology. In this review, we discuss on our current understanding of miR-155 function in virus-induced lymphomas focusing primarily on avian oncogenic viruses.

## 1. Introduction

Oncogenic viruses are associated with ~20% of cancers in humans, while in commercial livestock species such as poultry, they account for more than 90% of neoplastic diseases. Oncogenic viruses have contributed significantly to our understanding of some of the most important molecular mechanisms in cancer biology. For example, the identification of the first oncogene v-*src* in Rous sarcoma virus and v-*myc* in the avian retrovirus strain MC-29, contributed to the discovery of the growing family of oncogenes [1,2,3]. Similarly, it was the interaction of the multifunctional oncogenic virus-encoded proteins (e.g., SV40 large T antigen) that led to the discovery of the family of tumor-suppressor proteins such as the p53 and retinoblastoma, which gave further insights into the dynamics of neoplastic transformation and cell proliferation [4]. While most of the studies in functional cancer biology were focused on the role of virus- and host-encoded protein-coding genes, more recent research has identified the importance of noncoding RNAs, particularly the different populations of microRNAs (miRNAs) acting as regulators of major tumor pathways.

MiRNAs are a large class of 21–22 nucleotide RNAs that direct the RNA-induced silencing complex (RISC) typically within the 3′ untranslated regions of their target transcripts inhibiting their translation [5]. Generally located within the intronic or noncoding regions of the genome, they occur either as single unit or as miRNA clusters processed from polycistronic transcripts. With the potential to regulate multiple mRNA targets, miRNA networks are predicted to regulate about one-third of the mRNA transcripts in mammals [6]. Given the established roles of miRNAs in various aspects of the cellular regulatory machinery, it is not surprising that they are associated directly with many cancers [7]. In fact, miRNA biogenesis pathway itself can be adversely affected in several cancers due to multiple causes. These include aberrations in the genome, epigenetic modifications, virus-induced changes, metabolic imbalances, oxidative disequilibrium, dysregulation of oncogenes, and mutations in tumor suppressor genes. Such changes can lead to defective microprocessor proteins that can affect different steps in miRNA biogenesis from transcriptional initiation of pri-miRNA to the formation of functional mature miRNA. Many cancer-associated miRNAs are located in fragile sites, minimal regions of loss of heterozygosity, amplification, and breakpoint regions of the genome implicating them further in oncogenesis [8]. There is growing evidence to indicate that disruption of miRNA expression correlates with different types of human malignancies involving multiple tissues [9]. Oncogenic mutations in genes, such as XPO5, PACT, Ago1, and Ago2, as well as the deregulation of several other important microprocessor proteins such as the SMAD family, BMP, TGF-beta, DDX5, and DDX17 in different cancers also severely impact miRNA biogenesis pathways [10]. Furthermore, in infections with retroviruses such as HTLV-1, significant downregulation of Drosha protein that is required for the cleavage of pri-miRNA biogenesis can affect miRNA pathways [11]. However in most cancers, miRNAs function directly either as oncogenes or tumor-suppressors modulating critical genes associated with cancer. Nevertheless, accurate prediction of the oncogenic or tumor-suppressive role of different miRNAs in cancer is very difficult mainly because of the vast quantity and diversity of targets of any miRNA. The complexity of miRNA-mediated regulation is also influenced by the functions of the downstream mRNA targets, the levels of miRNA expression and the degree of miRNA-mediated inhibition, which are known to vary in the tissue-specific context [12]. As a result, many miRNAs such as miR-125b [13], miR-29 family [14], and miR-375 [15] can have tumor promotion as well as tumor-suppressor roles depending on different contexts. Based on the extensive studies identifying the plethora of miRNAs and their targets, it has become very clear that miRNAs participate extensively in modulating the altered transcriptome and proteome of the cancer cell, although their wider effects on the whole regulatory networks of the cell remain to be explored [16,17]. This is particularly significant in many cancer cells where widespread mRNA 3′ UTR shortening through alternative polyadenylation to escape miRNA-mediated repression leading to the disruption of competing endogenous RNA (ceRNA) crosstalk [17,18]. While a number of miRNAs have been associated with cancer, miRNA-155 (miR-155) and its functional orthologs have been extensively studied across many types of tumours [19,20] in different host species including poultry. In this review, we highlight the significant role of miR-155 and its virus-encoded orthologs in neoplastic transformation by avian oncogenic viruses.

## 2. MicroRNA-155

**Figure 1:**
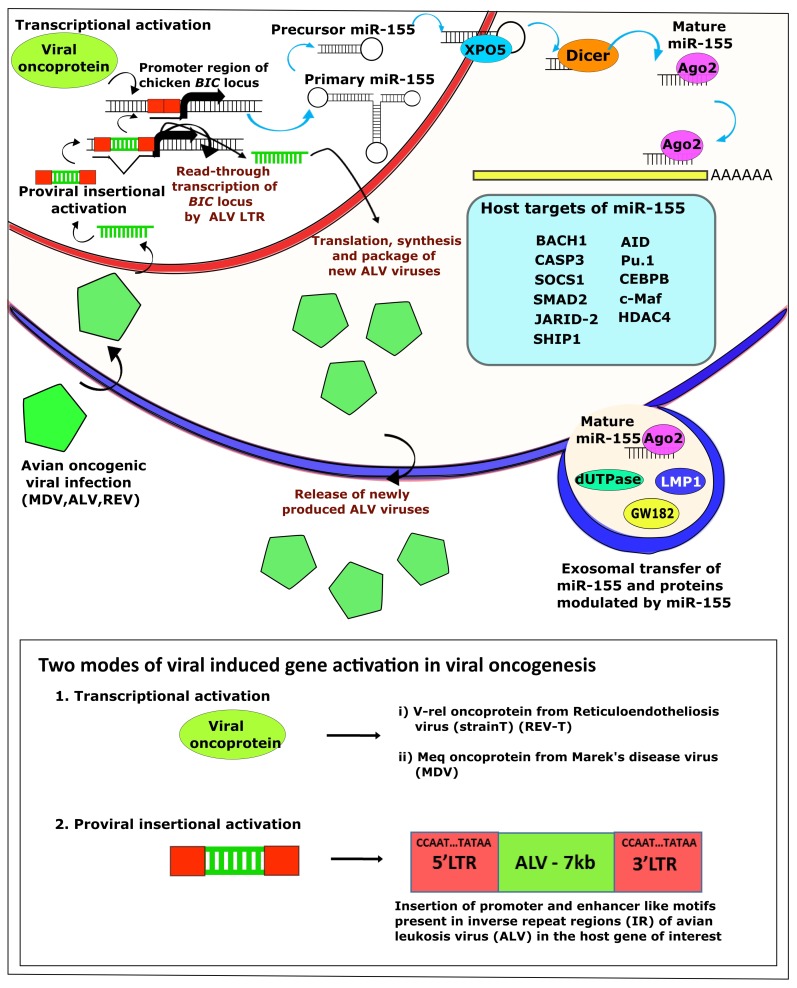
Molecular mechanisms of transformation by oncogenic viruses through miR-155 pathways. Induction of miR-155 through proviral insertional activation or through virus-encoded/transduced oncoproteins such as v-*myc*, v-*rel*, and Meq results in the modulation of expression of a number of miR-155 target proteins, contributing to the neoplastic transformation of the infected cell. Exosomal transfer of miR-155 and other proteins from the transformed cells can also contribute to the transformation of adjacent new cells.

The story of the multifunctional miRNA-155 begins with the studies on lymphomas in chickens infected with the highly oncogenic avian leukosis virus (ALV), where retroviral insertions in the oncogenes, such as c-*myc* and c-*myb*, were primarily associated with the neoplastic transformation [2]. An additional integration site identified in many of the ALV-induced lymphomas was named *bic* (for B cell integration cluster), which was associated with oncogene activation and tumor metastasis [2]. Although miRNAs were not discovered at that time, identification of the ALV-induced insertional activation of the *bic* gene that encoded a noncoding RNA was important, as it was later shown to be the precursor hairpin encoding miR-155 [21]. Subsequent extensive studies have demonstrated the association of miR-155 with different lymphomas [22,23,24] (Figure 1).

Such observations on the association of miR-155 and cancer were supported by the studies on transgenic mice overexpressing miR-155 which showed development of B cell lymphomas with downregulation of target proteins such as SHIP1 and CEBPβ [25,26]. MiR-155 is also involved in a number of other functions. For example, miR-155 has important role in immune responses both in the activation and differentiation of different cell types [12] particularly through its regulation of the inducible target gene *SOCS*1 [27]. Similarly, miR-155 has also been shown to be required for B cell responses to different antigens through its targeting of Pu.1 transcription factor that activates gene expression during plasma cell development with additional effects on AID-mediated gene conversion [28]. Despite these critical roles, miR-155 is predominantly known for their oncogenic roles [22,29].

## 3. Modulation of miR-155 by Oncogenic Viruses

**Figure 2:**
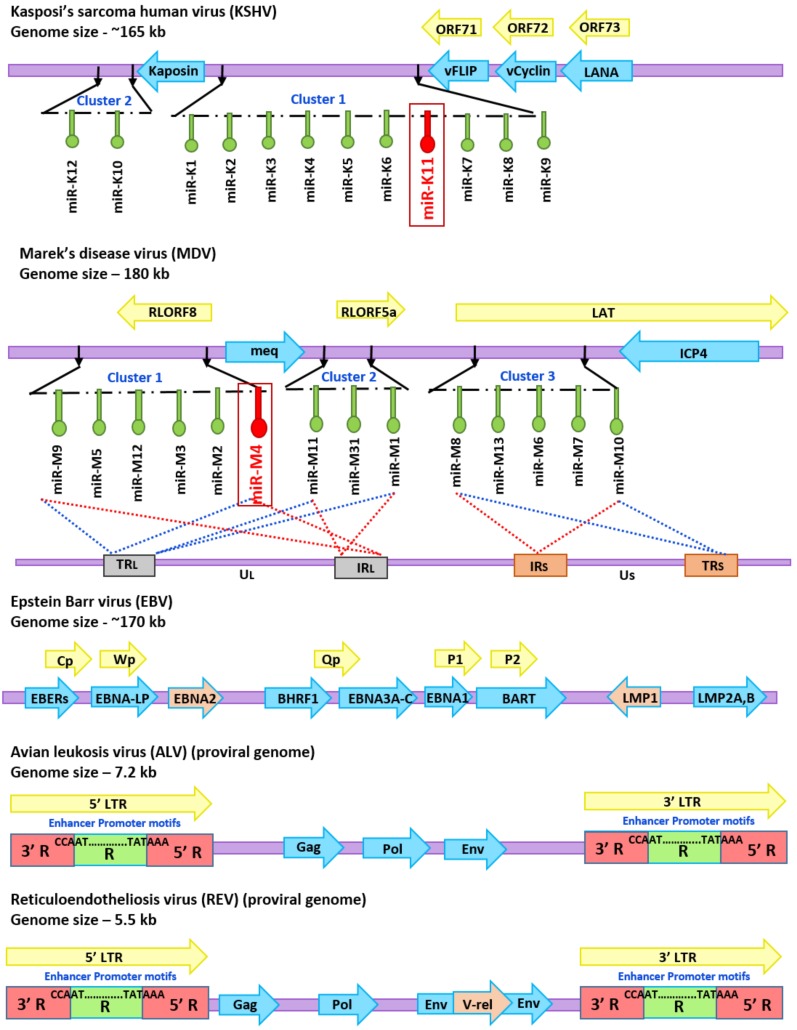
Diagrammatic representation of the genome structure of oncogenic viruses. MDV genome structure showing the locations of Meq and miRNAs including the miR-155 homolog miR-M4. KSHV genome structure with positions of miRNAs including the miR-155 homolog miR-K11 is shown for comparison. EBV genome showing the positions of genes such as EBNA2 and LMP1 that can modulate the expression of miR-155 are shown. Provirus structures of ALV with the 5′ and 3′ LTR involved in the insertional activation of *bic/*miR-155 and REV with the transduced v-*rel* oncogene are also shown.

With the ability to use multiple strategies for inducing rapid neoplastic transformation of the target cell types, it was never surprising that oncogenic viruses also exploited the miRNA machinery. Intervention of the miRNA pathway by the oncogenic viruses occurs mainly through the modulation of the expression of the existing cellular miRNAs as shown in the miRNA expression profiling studies of different virus-transformed cells [30,31]. In addition, a number of oncogenic viruses have also evolved to encode their own miRNAs arming them with the capacity to modulate the expression of hundreds of host genes [31,32,33]. However, there are differences between virus families in making use of the miRNA pathway in regulating gene expression. Herpesviruses are the most successful among different viruses to exploit the miRNA pathway, perhaps due to their distinct style of virus–host interactions with lifelong persistence in the host as latent infections [31,34]. Some viruses also encode functional orthologs of the already existing host miRNAs, enabling them to modulate the expression of overlapping set of target genes. Best example of the virus-encoded miRNA ortholog comes from the Kaposi’s sarcoma herpesvirus (KSHV), a human gammaherpesvirus associated with lymphoproliferative disorders such as primary effusion lymphoma (PEL), multicentric Castleman disease (MCD), and B lymphomagenesis in AIDS patients. In addition to a plethora of viral proteins and host protein orthologs, KSHV encodes 25 miRNAs many of which play important roles in lymphoproliferation and viral pathogenesis. Among these miRNAs, KSHV-K12-11 that plays critical role in pathogenesis is a functional ortholog of hsa-miR-155 sharing identical seed sequences [35,36,37]. Photoactivatable ribonucleoside-enhanced crosslinking and immunoprecipitation (PAR-CLIP) analysis discovered >300 putative mRNA targets for KSHV-K12-11, which overlapped with that of the host has-miR-155. This included SMAD1, SMAD2, SMAD3, and SMAD5, which were confirmed as potential miR-155 targets by reporter assays [38]. More recent studies have identified a number of novel targets of KSHV-K12-11 that involved processes related to oncogenesis, such as glycolysis, apoptosis, and cell cycle control [39]. We have since shown that Marek’s disease virus (MDV-1), a herpesvirus associated with T-cell lymphoma in chickens, also encoded mdv1-miR-M4-5p (miR-M4), another functional ortholog of miR-155 that plays a critical role in pathogenesis [40] (Figure 2).

**Figure 3:**
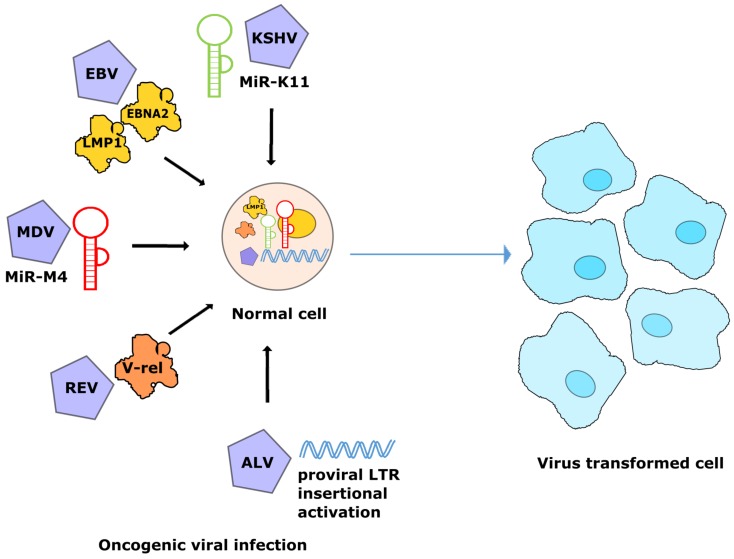
Conserved mechanisms of transformation by oncogenic viruses using miR-155 pathway. Expression of viral homologs of miR-155 (MDV-miRM4, and KSHV-miR-K11), activation of miR-155 expression through virus-encoded/transduced oncoproteins (EBV-encoded EBNA2, LMP1, and REV-transduced v-*rel*) and direct insertional activation of *bic* gene by avian leukosis virus (ALV) provirus.

High level expression of these viral homologs in the infected cells allows KSHV and MDV-1 for efficient modulation of the miR-155 targetome [41,42] including major hematopoietic targets such as Pu.1, BACH-1, and AID. Epstein–Barr virus (EBV), another oncogenic herpesvirus associated with B-cell lymphomas, including Hodgkin’s lymphoma, diffuse large B cell lymphoma (DLBCL), and Burkitt’s lymphoma in humans, upregulates miR-155 resulting in escalated cell proliferation and neoplastic transformation [43,44]. This strategic maneuvering of the host miR-155 pathway to induce transformation is accomplished through the effects of two EBV-encoded proteins LMP1 and EBNA-2 (Figure 2). Interestingly EBV harbors 25 miRNA precursors encoding at least 49 mature miRNAs. However, unlike KSHV and MDV-1 [9], none of these are homologs of miR-155 and have no conservation of seed sequences. Thus, miR-155 pathway appears to be important for pathogenesis and neoplastic transformation by these oncogenic viruses (Figure 3). However, it is less clear why EBV has opted to overexpress the host miR-155, while KSHV and MDV-1 have adopted the strategy to encode their own homologs.

## 4. Avian Oncogenic Viruses and miR-155

### 4.1. MDV-1 and miRNAs

Marek’s disease (MD), caused by MDV-1, is a lymphoproliferative disease of chickens characterized by rapid-onset lymphomas in multiple organs and infiltration into peripheral nerves causing paralysis. MD is widespread in the poultry population around the world with estimated economic losses of up to US$ 2 billion annually. MDV-1, is an alphaherpesvirus belonging to the Mardivirus genus, that also include the nonpathogenic Gallid herpesvirus 3 and the antigenically related herpesvirus of turkey (HVT), both of which are used as vaccines and viral vectors. MDV-1 itself includes different isolates varying in virulence ranging from CVI988 (Rispens) vaccine strain to the virulent (vMDV), very virulent (vvMDV), and very virulent plus (vv+MDV) pathotypes. Despite the use of widespread vaccination strategy for nearly 5 decades using different vaccine strains, MDV-1 strains show continuing evolution of virulence [45].

Evidence of MDV-1-encoded miRNAs was first obtained from chicken embryo fibroblast (CEF) infected with the highly virulent RB1B strain of MDV-1 [46], followed by the analysis of small RNA library from MSB-1, a lymphoblastoid cell line established from an MD lymphoma of the spleen [47,48]. This included 14 miRNA precursors producing 26 mature miRNAs clustered into three separate genomic loci [46,47,48] within the inverted repeat regions of the genome. There is high degree of sequence conservation among different MDV-1 strains. However, differences in the expression levels have been observed between strains of varying virulence [48,49], particularly those which are expressed from the cluster 1 [50,51], implying that these miRNAs may have a more significant role in MD oncogenesis. We have since shown that this was indeed the case, as the deletion of the cluster 1 miRNAs abolished the oncogenicity of the virulent RB-1B strain [52]. Our study also revealed that miR-M4 that accounted for more than two-thirds of the miRNA reads in tumor cells, is the single most important miRNA in this cluster that contributed to oncogenesis [47,51]. Further studies have shown that a single promoter, that is kept epigenetically active by activating histone marks and hypomethylation [53] drives the expression of miRNAs in the clusters 1 and 2 [50]. Discovery of miR-M4 as a functional homolog of miR-155 with the capability of regulating many of the targets of miR-155 [40,51] further highlighted its importance in lymphocyte transformation by MDV as well as other oncogenic herpesviruses such as EBV and KSHV that also used miR-155 regulatory pathway. Comparison of the targets of hsa-miR-155, KSHV-miR-K12-11, and MDV-miR-M4 in KSHV- or EBV-transformed human B cells and MDV-1-transformed MSB-1 cells, respectively, showed that nine common targets were shared by all the three miR-155 orthologs [41]. Four additional targets were shown to be conserved among the EBV-induced hsa-miR-155 and MDV-miR-M4 [41]. Some of these shared targets such as JARID-2 (a histone methyltransferase that promote apoptosis decrease cell survival), and NF-κB-inducing kinase could be expected to regulate lymphocyte biology across the species. Thus, the conservation in the targeting of the 3′UTRs of some of these genes by miR-155 seed family members demonstrate the importance of these regulatory pathways between humans and chickens, despite their separation by evolutionary distance of ~300 million years [41].

High level expression of the miR-155 homolog MDV-miR-M4 in tumor cells and the identification of several target proteins related to the cancer pathway strongly suggested an important role for this miRNA in virus-induced lymphomagenesis. However, the best way to confirm this important role is to examine the role of mutant viruses. The combination of the availability of excellent disease models in the natural chicken hosts and reverse genetics tools for manipulating the MDV genome, provide the perfect opportunity to examine their roles in natural infection models. Using viruses modified by reverse genetics of the infectious BAC clone of the oncogenic RB-1B strain of MDV, we showed that the deletion of the six-miRNA cluster 1 from the viral genome abolished the oncogenicity of the virus [52,54], and the loss of oncogenicity appeared to be primarily due to miR-M4 in the cluster. This was the first demonstration of the direct in vivo role of a virus-encoded miRNA in inducing tumors in a natural infection model, since the deletion or a 2-nucleotide mutation within its seed region was sufficient to inhibit the induction of lymphomas. The definitive role of this miR-155 ortholog in oncogenicity was further confirmed by the rescue of oncogenic phenotype by revertant viruses that expressed either the miR-M4 or the cellular homolog gga-miR-155 [52]. Despite its importance in the induction of MD lymphomas, our preliminary data from MDV-transformed cell lines suggest that continued expression of miR-M4 is not essential to maintain the transformed phenotype (unpublished data).

### 4.2. ALV and miRNAs

Avian leukosis includes several different leukemia-like neoplastic diseases of the hematopoietic system in poultry. These tumors are induced by avian leukosis viruses (ALV), members of the alpharetrovirus genus of the subfamily orthoretrovirinae in the family Retroviridae. ALV genome has, from the 5′ end to the 3′ end, three structural genes—gag/pro-pol-env—which encode the proteins of the virion group-specific (gs) antigens and protease, the reverse transcriptase enzyme, and the envelope glycoproteins, respectively. Based on the properties of the envelope glycoproteins, ALVs are grouped into different subgroups that could be differentiated based on their host range, interference and neutralization profiles. As with other retroviruses, the ALV life cycle involves multiple steps starting with specific receptor binding and entry into the cell, reverse transcription, and integration of the proviral DNA into the host genome, and production of new viruses that are released from the infected cell. Induction of tumors by ALV occurs by insertional mutagenesis, where the integration of ALV activates cellular proto-oncogenes, such as c-*myc* and c-*erb*B, causing lymphoid, myeloid, or erythroid tumors depending on the affected hematopoietic cells [55].

In addition to the activation of a number of well-characterized oncogenes, ALVs have also been successful in modulating cellular miRNA pathway, as is evident from our study where we showed altered expression of a number of miRNAs in ALV-transformed cell lines such as DT40 and HD11 [56]. More importantly, the activation of miR-155, first identified as the product of the *bic* gene was shown to be frequently upregulated in B-cell lymphoma in chickens [2,57]. Although it is possible that such changes in miRNAs are secondary to the expression of the activated oncoproteins, it is clear that ALVs use miRNA pathway to maintain the transformed phenotype of the tumour cells. Unlike many herpesviruses that encode multiple miRNAs [31], retroviruses are not generally known to encode the virus-encoded miRNAs. However, using a deep-sequencing approach on one of the ALV-J-transformed cell lines, we identified a small RNA encoded within the E (XSR) element of the ALV-J genome [58]. Although the biological significance or targets of this novel miRNA remain to be identified, their high expression in ALV-J-transformed cell lines, such as IAH30, suggests a functional role either in viral replication or in transformation.

A diverse number of functions have been associated with miR-155, ranging from regulation of the immune responses to inflammation to the myeloid lineage specification [12]. As a powerful oncomiRNA, it is an important driver of B cell lymphoma. However, ALV integration in other specific genes such as c-myc, c-myb, and TERT are common and potentially function as powerful oncogenic determinants [59]. Delineating the molecular pathways associated with the insertional activation of miR-155 from that of other oncogenes will be important to understand the precise roles of miR-155 in ALV-induced transformation. Although activation of miR-155 may have an important role in ALV-induced transformation, this appears to be not necessary in all cases. For example DT-40, a transformed chicken B cell line derived from ALV-induced B-cell lymphomas, did not show increased miR-155 expression [56]. Furthermore, we have shown that CRISPR/Cas9-mediated deletion of miR-155 from ALV-transformed cell line HP45 did not affect the transformed phenotype as demonstrated by the continued proliferation of the miR-155-knockout cell line (unpublished).

### 4.3. REV and miRNAs

Reticuloendotheliosis refers to a group of syndromes in poultry and game birds associated with reticuloendotheliosis virus (REV), another virus belonging to the family Retroviridae. Syndromes associated with REV include the runting disease syndrome characterized by runting, bursal, and thymic atrophy, enlarged peripheral nerves, abnormal feather development, proventriculitis, enteritis, anemia, and liver and spleen necrosis [60]. An accidental contamination of a plasmodium strain injected into chickens has been suggested as a possible origin of this virus [61]. REV is also known for inducing chronic lymphoid neoplasms in the bursa of Fabricius and other organs, usually by the insertional activation of the c-myc oncogene. Nonbursal lymphomas of T cell origin involving multiple organs such as the thymus, liver, heart and spleen have also been induced in birds experimentally infected with REV. An acutely transforming variant of the virus REV-T carrying the transduced oncogene v-rel, a member of the rel/NF-kB family of transcription factors, can induce rapid transformation of primary avian hematopoietic cells [60]. The v-rel-mediated transformation occurs predominantly through the modulation of rel/NF-kB targets. We and others have shown that v-rel-induced transformation is accompanied by increased expression of miRNA-155 triggered through the direct binding to the putative NF-kB-binding sites in the *bic* promoter [31,62]. Analysis of the transcriptome changes in REV-T- transformed chicken B cells identified 73 genes with predicted miR-155 target sites, the top hit of the most enriched miRNA targets [63]. Analysis of the expression profiles of avian cells overexpressing miR-155 have identified the downregulation of several miR-155 target proteins resulting in the modulation of major functional pathways, including cancer-related pathways, supporting the direct role of miR-155 in neoplastic transformation [63]. Interestingly, EBV-encoded latent membrane protein-1, a potent inducer of miR-155, also targets the NF-kB-binding sites in the *bic* promoter. Coincidentally, upregulation of c-myc as well as miR-155 were observed in REV-transformed bursal and splenic tissues. The functional similarity of v-rel to NF-kB transcription factor suggested that the protein can ideally be a potential regulator and partner required for downstream pathways initiated by miR-155.

## 5. Discussion and Conclusions

Neoplastic transformation is a multistep process involving complex molecular events leading to the generation of transformed cancer cells with phenotypes such as uncontrolled proliferation, evasion of antigrowth signals and apoptosis, replicative immortality, metastatic capability, and many others. Oncogenic viruses, as very efficient inducers to cancer, have mastered the ability for critical manipulation of the regulatory pathways that are needed for transforming a normal cell into a cancer cell. Most oncogenic viruses maintain a persistent infection hidden from the immune system and cause deregulated expression of oncogenes and/or tumor-suppressor genes to induce transformation. While protein coding genes were considered the major oncogenic determinants until recently, a number of noncoding RNAs especially miRNAs, are also increasingly being recognized as major determinants. Among the miRNAs identified for their association with cancer, miR-155 is very important in terms of its widespread involvement in multiple tumors and in the modulation of a wide spectrum of target proteins. Moreover, exploitation of the miR-155 pathway by multiple oncogenic viruses affecting different species further strengthens the direct role of this important oncogenic miRNA. Oncogenic human viruses EBV and KSHV both use the miR-155 pathway to regulate the transformation process, albeit by independent mechanisms [30]. Similarly avian oncogenic viruses MDV, ALV, and REV, all of which are efficient inducers of transformation of avian hematopoietic cells, appears to converge in using the miR-155 pathway in achieving neoplastic transformation [31].

Recent new insights on the operational landscape of miRNAs in regulating gene circuits in relation to their abundances and their targets [64] will further enhance our knowledge on miR-155 and its virus-encoded homologs, which are usually expressed at very high levels in virus-induced cancer cells. In addition, recent demonstration that miR-155 as a key driver regulator of sustained exhausted T cell responses during chronic infections by pathogens such as lymphocytic choriomeningitis virus [65] can have implications in virus-induced cancers. Exosomal transfer of functional miRNAs between cells is an emerging field which can help in assigning functional contributions of miRNAs in cell to cell communication, especially in cancer signaling. It has been demonstrated that miR-155 packaged in exosomes from cancer cells can be delivered to other cell types to confer resistance to chemotherapeutic agents [66]. Interestingly, the exosomes also play important roles in cell-to-cell communication of virus-transformed cells. For example, EBV-mediated transfer of BART and BHRF1-associated virus-encoded miRNAs from cancerous cells to noncancerous cells through exosomes have been demonstrated [67]. Recent study has also suggested the opportunities of using the serum exosome-associated miRNAs as potential biomarkers for MD tumors [68]. Finally, there is increasing evidence of 3′UTR shortening and alternative polyadenylation of transcripts in cancer which also will have significant impact on miRNA-mediated gene regulation [18,69]. It would be interesting to examine whether viruses might also exploit such mechanisms for neoplastic transformation. Avian oncogenic viruses and the tumor disease models can be valuable in examining the intricacies of miRNA functions, especially that of miR-155, as we are beginning to unearth more details of the avian genome and its regulatory landscape.

## References

[1] Weiss R.A., Vogt P.K. (2011). 100 years of Rous sarcoma virus. J. Exp. Med..

[2] Clurman B.E., Hayward W.S. (1989). Multiple proto-oncogene activations in avian leukosis virus-induced lymphomas: Evidence for stage-specific events. Mol. Cell. Biol..

[3] Rubin H. (2011). The early history of tumor virology: Rous, RIF, and RAV. Proc. Natl. Acad. Sci. USA.

[4] Tornesello M.L., Annunziata C., Tornesello A.L., Buonaguro L., Buonaguro F.M. (2018). Human Oncoviruses and p53 Tumor Suppressor Pathway Deregulation at the Origin of Human Cancers. Cancers.

[5] Bartel D.P. (2004). MicroRNAs: Genomics, biogenesis, mechanism, and function. Cell.

[6] Filipowicz W., Bhattacharyya S.N., Sonenberg N. (2008). Mechanisms of post-transcriptional regulation by microRNAs: Are the answers in sight?. Nat. Rev. Genet..

[7] Volinia S., Galasso M., Costinean S., Tagliavini L., Gamberoni G., Drusco A., Marchesini J., Mascellani N., Sana M.E., Abu Jarour R. (2010). Reprogramming of miRNA networks in cancer and leukemia. Genome Res..

[8] Calin G.A., Sevignani C., Dumitru C.D., Hyslop T., Noch E., Yendamuri S., Shimizu M., Rattan S., Bullrich F., Negrini M. (2004). Human microRNA genes are frequently located at fragile sites and genomic regions involved in cancers. Proc. Natl. Acad. Sci. USA.

[9] Lin Z., Flemington E.K. (2011). miRNAs in the pathogenesis of oncogenic human viruses. Cancer Lett..

[10] Lin S., Gregory R.I. (2015). MicroRNA biogenesis pathways in cancer. Nat. Rev. Cancer.

[11] Van Duyne R., Guendel I., Klase Z., Narayanan A., Coley W., Jaworski E., Roman J., Popratiloff A., Mahieux R., Kehn-Hall K. (2012). Localization and sub-cellular shuttling of HTLV-1 tax with the miRNA machinery. PLoS ONE.

[12] Hsin J.P., Lu Y., Loeb G.B., Leslie C.S., Rudensky A.Y. (2018). The effect of cellular context on miR-155-mediated gene regulation in four major immune cell types. Nat. Immunol..

[13] Svoronos A.A., Engelman D.M., Slack F.J. (2016). OncomiR or Tumor Suppressor? The Duplicity of MicroRNAs in Cancer. Cancer Res..

[14] Pekarsky Y., Croce C.M. (2010). Is miR-29 an oncogene or tumor suppressor in CLL?. Oncotarget.

[15] Costa-Pinheiro P., Ramalho-Carvalho J., Vieira F.Q., Torres-Ferreira J., Oliveira J., Goncalves C.S., Costa B.M., Henrique R., Jeronimo C. (2015). MicroRNA-375 plays a dual role in prostate carcinogenesis. Clin. Epigenet..

[16] Gebert L.F.R., MacRae I.J. (2018). Regulation of microRNA function in animals. Nat. Rev. Mol. Cell Biol..

[17] Anastasiadou E., Jacob L.S., Slack F.J. (2018). Non-coding RNA networks in cancer. Nat. Rev. Cancer.

[18] Park H.J., Ji P., Kim S., Xia Z., Rodriguez B., Li L., Su J., Chen K., Masamha C.P., Baillat D. (2018). 3’ UTR shortening represses tumor-suppressor genes in trans by disrupting ceRNA crosstalk. Nat. Genet..

[19] Due H., Svendsen P., Bodker J.S., Schmitz A., Bogsted M., Johnsen H.E., El-Galaly T.C., Roug A.S., Dybkaer K. (2016). miR-155 as a Biomarker in B-Cell Malignancies. Biomed. Res. Int..

[20] Narayan N., Bracken C.P., Ekert P.G. (2018). MicroRNA-155 expression and function in AML: An evolving paradigm. Exp. Hematol..

[21] Tam W. (2001). Identification and characterization of human BIC, a gene on chromosome 21 that encodes a noncoding RNA. Gene.

[22] Eis P.S., Tam W., Sun L., Chadburn A., Li Z., Gomez M.F., Lund E., Dahlberg J.E. (2005). Accumulation of miR-155 and BIC RNA in human B cell lymphomas. Proc. Natl. Acad. Sci. USA.

[23] Kluiver J., Poppema S., de Jong D., Blokzijl T., Harms G., Jacobs S., Kroesen B.J., van den Berg A. (2005). BIC and miR-155 are highly expressed in Hodgkin, primary mediastinal and diffuse large B cell lymphomas. J. Pathol..

[24] Van den Berg A., Kroesen B.J., Kooistra K., de Jong D., Briggs J., Blokzijl T., Jacobs S., Kluiver J., Diepstra A., Maggio E. (2003). High expression of B-cell receptor inducible gene BIC in all subtypes of Hodgkin lymphoma. Genes Chromosomes Cancer.

[25] Costinean S., Sandhu S.K., Pedersen I.M., Tili E., Trotta R., Perrotti D., Ciarlariello D., Neviani P., Harb J., Kauffman L.R. (2009). Src homology 2 domain-containing inositol-5-phosphatase and CCAAT enhancer-binding protein beta are targeted by miR-155 in B cells of Emicro-MiR-155 transgenic mice. Blood.

[26] Costinean S., Zanesi N., Pekarsky Y., Tili E., Volinia S., Heerema N., Croce C.M. (2006). Pre-B cell proliferation and lymphoblastic leukemia/high-grade lymphoma in E(mu)-miR155 transgenic mice. Proc. Natl. Acad. Sci. USA.

[27] Lu L.F., Gasteiger G., Yu I.S., Chaudhry A., Hsin J.P., Lu Y., Bos P.D., Lin L.L., Zawislak C.L., Cho S. (2015). A Single miRNA-mRNA Interaction Affects the Immune Response in a Context- and Cell-Type-Specific Manner. Immunity.

[28] Vigorito E., Perks K.L., Abreu-Goodger C., Bunting S., Xiang Z., Kohlhaas S., Das P.P., Miska E.A., Rodriguez A., Bradley A. (2007). microRNA-155 regulates the generation of immunoglobulin class-switched plasma cells. Immunity.

[29] Seddiki N., Brezar V., Ruffin N., Levy Y., Swaminathan S. (2014). Role of miR-155 in the regulation of lymphocyte immune function and disease. Immunology.

[30] Vojtechova Z., Tachezy R. (2018). The Role of miRNAs in Virus-Mediated Oncogenesis. Int. J. Mol. Sci..

[31] Yao Y., Nair V. (2014). Role of virus-encoded microRNAs in Avian viral diseases. Viruses.

[32] Fan C., Tang Y., Wang J., Xiong F., Guo C., Wang Y., Xiang B., Zhou M., Li X., Wu X. (2018). The emerging role of Epstein-Barr virus encoded microRNAs in nasopharyngeal carcinoma. J. Cancer.

[33] Kincaid R.P., Sullivan C.S. (2012). Virus-encoded microRNAs: An overview and a look to the future. PLoS Pathog..

[34] Piedade D., Azevedo-Pereira J.M. (2016). The Role of microRNAs in the Pathogenesis of Herpesvirus Infection. Viruses.

[35] Gottwein E., Mukherjee N., Sachse C., Frenzel C., Majoros W.H., Chi J.T., Braich R., Manoharan M., Soutschek J., Ohler U. (2007). A viral microRNA functions as an orthologue of cellular miR-155. Nature.

[36] Skalsky R.L., Samols M.A., Plaisance K.B., Boss I.W., Riva A., Lopez M.C., Baker H.V., Renne R. (2007). Kaposi’s sarcoma-associated herpesvirus encodes an ortholog of miR-155. J. Virol..

[37] Sin S.H., Kim Y.B., Dittmer D.P. (2013). Latency locus complements MicroRNA 155 deficiency in vivo. J. Virol..

[38] Liu Y., Sun R., Lin X., Liang D., Deng Q., Lan K. (2012). Kaposi’s sarcoma-associated herpesvirus-encoded microRNA miR-K12-11 attenuates transforming growth factor beta signaling through suppression of SMAD5. J. Virol..

[39] Gay L.A., Sethuraman S., Thomas M., Turner P.C., Renne R. (2018). Modified Cross-Linking, Ligation, and Sequencing of Hybrids (qCLASH) Identifies Kaposi’s Sarcoma-Associated Herpesvirus MicroRNA Targets in Endothelial Cells. J. Virol..

[40] Zhao Y., Yao Y., Xu H., Lambeth L., Smith L.P., Kgosana L., Wang X., Nair V. (2009). A functional MicroRNA-155 ortholog encoded by the oncogenic Marek’s disease virus. J. Virol..

[41] Parnas O., Corcoran D.L., Cullen B.R. (2014). Analysis of the mRNA targetome of microRNAs expressed by Marek’s disease virus. mBio.

[42] Gottwein E., Corcoran D.L., Mukherjee N., Skalsky R.L., Hafner M., Nusbaum J.D., Shamulailatpam P., Love C.L., Dave S.S., Tuschl T. (2011). Viral microRNA targetome of KSHV-infected primary effusion lymphoma cell lines. Cell Host Microbe.

[43] Linnstaedt S.D., Gottwein E., Skalsky R.L., Luftig M.A., Cullen B.R. (2010). Virally induced cellular microRNA miR-155 plays a key role in B-cell immortalization by Epstein-Barr virus. J. Virol..

[44] Wood C.D., Carvell T., Gunnell A., Ojeniyi O.O., Osborne C., West M.J. (2018). Enhancer control of miR-155 expression in Epstein-Barr virus infected B cells. J. Virol..

[45] Read A.F., Baigent S.J., Powers C., Kgosana L.B., Blackwell L., Smith L.P., Kennedy D.A., Walkden-Brown S.W., Nair V.K. (2015). Imperfect Vaccination Can Enhance the Transmission of Highly Virulent Pathogens. PLoS Biol..

[46] Burnside J., Bernberg E., Anderson A., Lu C., Meyers B.C., Green P.J., Jain N., Isaacs G., Morgan R.W. (2006). Marek’s disease virus encodes MicroRNAs that map to meq and the latency-associated transcript. J. Virol..

[47] Yao Y., Zhao Y., Xu H., Smith L.P., Lawrie C.H., Watson M., Nair V. (2008). MicroRNA profile of Marek’s disease virus-transformed T-cell line MSB-1: Predominance of virus-encoded microRNAs. J. Virol..

[48] Morgan R., Anderson A., Bernberg E., Kamboj S., Huang E., Lagasse G., Isaacs G., Parcells M., Meyers B.C., Green P.J. (2008). Sequence conservation and differential expression of Marek’s disease virus microRNAs. J. Virol..

[49] Burnside J., Morgan R. (2011). Emerging roles of chicken and viral microRNAs in avian disease. BMC Proc..

[50] Coupeau D., Dambrine G., Rasschaert D. (2012). Kinetic expression analysis of the cluster mdv1-mir-M9-M4, genes meq and vIL-8 differs between the lytic and latent phases of Marek’s disease virus infection. J. Gen. Virol..

[51] Zhuang G., Sun A., Teng M., Luo J. (2017). A Tiny RNA that Packs a Big Punch: The critical role of a viral miR-155 ortholog in lymphomagenesis in Marek’s disease. Front. Microbiol..

[52] Zhao Y., Xu H., Yao Y., Smith L.P., Kgosana L., Green J., Petherbridge L., Baigent S.J., Nair V. (2011). Critical role of the virus-encoded microRNA-155 ortholog in the induction of Marek’s disease lymphomas. PLoS Pathog..

[53] Brown A.C., Nair V., Allday M.J. (2012). Epigenetic regulation of the latency-associated region of Marek’s disease virus in tumor-derived T-cell lines and primary lymphoma. J. Virol..

[54] Yu Z.H., Teng M., Sun A.J., Yu L.L., Hu B., Qu L.H., Ding K., Cheng X.C., Liu J.X., Cui Z.Z. (2014). Virus-encoded miR-155 ortholog is an important potential regulator but not essential for the development of lymphomas induced by very virulent Marek’s disease virus. Virology.

[55] Nair V., Fadly A., Swayne D.E., Glisson J.R., McDougald L.R., Nolan L.K., Suarez D.L., Nair V. (2013). Leukosis/Sarcoma group. Diseases of Poultry.

[56] Yao Y., Charlesworth J., Nair V., Watson M. (2013). MicroRNA expression profiles in avian haemopoietic cells. Front. Genet..

[57] Tam W., Ben-Yehuda D., Hayward W.S. (1997). BIC, a novel gene activated by proviral insertions in avian leukosis virus-induced lymphomas, is likely to function through its noncoding RNA. Mol. Cell. Biol..

[58] Yao Y., Smith L.P., Nair V., Watson M. (2014). An avian retrovirus uses canonical expression and processing mechanisms to generate viral microRNA. J. Virol..

[59] Justice J.F., Morgan R.W., Beemon K.L. (2015). Common Viral Integration Sites Identified in Avian Leukosis Virus-Induced B-Cell Lymphomas. mBio.

[60] Nair V., Zavala G., Fadly A.M., Swayne D.E., Glisson J.R., McDougald L.R., Nolan L.K., Suarez D.L., Nair V. (2013). Reticuloendotheliosis. Diseases of Poultry.

[61] Niewiadomska A.M., Gifford R.J. (2013). The extraordinary evolutionary history of the reticuloendotheliosis viruses. PLoS Biol..

[62] Bolisetty M.T., Dy G., Tam W., Beemon K.L. (2009). Reticuloendotheliosis virus strain T induces miR-155, which targets JARID2 and promotes cell survival. J. Virol..

[63] Yao Y., Vasoya D., Kgosana L., Smith L.P., Gao Y., Wang X., Watson M., Nair V. (2017). Activation of gga-miR-155 by reticuloendotheliosis virus T strain and its contribution to transformation. J. Gen. Virol..

[64] Quarton T., Ehrhardt K., Lee J., Kannan S., Li Y., Ma L., Bleris L. (2018). Mapping the operational landscape of microRNAs in synthetic gene circuits. NPJ Syst. Biol. Appl..

[65] Stelekati E., Chen Z., Manne S., Kurachi M., Ali M.A., Lewy K., Cai Z., Nzingha K., McLane L.M., Hope J.L. (2018). Long-Term Persistence of Exhausted CD8 T Cells in Chronic Infection Is Regulated by MicroRNA-155. Cell Rep..

[66] Mikamori M., Yamada D., Eguchi H., Hasegawa S., Kishimoto T., Tomimaru Y., Asaoka T., Noda T., Wada H., Kawamoto K. (2017). MicroRNA-155 Controls Exosome Synthesis and Promotes Gemcitabine Resistance in Pancreatic Ductal Adenocarcinoma. Sci. Rep..

[67] Pegtel D.M., Cosmopoulos K., Thorley-Lawson D.A., van Eijndhoven M.A.J., Hopmans E.S., Lindenberg J.L., de Gruijl T.D., Würdinger T., Middeldorp J.M. (2010). Functional delivery of viral miRNAs via exosomes. Proc. Natl. Acad. Sci. USA.

[68] Nath Neerukonda S., Egan N.A., Patria J., Assakhi I., Tavlarides-Hontz P., Modla S., Munoz E.R., Hudson M.B., Parcells M.S. (2019). Comparison of exosomes purified via ultracentrifugation (UC) and Total Exosome Isolation (TEI) reagent from the serum of Marek’s disease virus (MDV)-vaccinated and tumor-bearing chickens. J. Virol. Methods.

[69] Passacantilli I., Panzeri V., Bielli P., Farini D., Pilozzi E., Fave G.D., Capurso G., Sette C. (2017). Alternative polyadenylation of ZEB1 promotes its translation during genotoxic stress in pancreatic cancer cells. Cell Death Dis..

